# High *Plasmodium malariae* Prevalence in an Endemic Area of the Colombian Amazon Region

**DOI:** 10.1371/journal.pone.0159968

**Published:** 2016-07-28

**Authors:** Paola Andrea Camargo-Ayala, Juan Ricardo Cubides, Carlos Hernando Niño, Milena Camargo, Carlos Arturo Rodríguez-Celis, Teódulo Quiñones, Lizeth Sánchez-Suárez, Manuel Elkin Patarroyo, Manuel Alfonso Patarroyo

**Affiliations:** 1 Molecular Biology and Immunology Department, Fundación Instituto de Inmunología de Colombia (FIDIC), Carrera 50 # 26–20, Bogotá, Colombia; 2 School of Medicine, Universidad Nacional de Colombia, Carrera 45 # 26–85, Bogotá, Colombia; 3 School of Medicine and Health Sciences, Universidad del Rosario, Carrera 24 # 63C-69, Bogotá, Colombia; 4 Gobernación del Amazonas, Calle 10 # 10–77, Leticia, Colombia; National Institutes of Health, UNITED STATES

## Abstract

Malaria is a worldwide public health problem; parasites from the genus *Plasmodium* are the aetiological agent for this disease. The parasites are mostly diagnosed by conventional microscopy-based techniques; however, their limitations have led to under-registering the reported prevalence of *Plasmodium* species. This study has thus been aimed at evaluating the infection and coinfection prevalence of 3 species of *Plasmodium* spp., in an area of the Colombian Amazon region. Blood samples were taken from 671 symptomatic patients by skin puncture; a nested PCR amplifying the 18S ssRNA region was used on all samples to determine the presence of *P*. *vivax*, *P*. *malariae* and *P*. *falciparum*. Statistical analysis determined infection and coinfection frequency; the association between infection and different factors was established. The results showed that *P*. *vivax* was the species having the greatest frequency in the study population (61.4%), followed by *P*. *malariae* (43.8%) and *P*. *falciparum* (11.8%). The study revealed that 35.8% of the population had coinfection, the *P*. *vivax*/*P*. *malariae* combination occurring most frequently (28.3%); factors such as age, geographical origin and clinical manifestations were found to be associated with triple-infection. The prevalence reported in this study differed from previous studies in Colombia; the results suggest that diagnosis using conventional techniques could be giving rise to underestimating some *Plasmodium* spp. species having high circulation rates in Colombia (particularly in the Colombian Amazon region). The present study’s results revealed a high prevalence of *P*. *malariae* and mixed infections in the population being studied. The results provide relevant information which should facilitate updating the epidemiological panorama and species’ distribution so as to include control, prevention and follow-up measures.

## Introduction

Malaria represents a public health problem for many countries around the world, being the main cause of morbidity and mortality for many of them. It has been estimated that 3,200 million people are at risk [1]; according to WHO reports for 2014, 214 million cases occurred, 438,000 of these resulting in death [2].

Protozoan parasites from the genus *Plasmodium* are the aetiological agent for this disease. Only 5 of the 200 species described to date can infect human beings: *P*. *falciparum*, *P*. *vivax*, *P*. *malariae*, *P*. *ovale* and *P*. *knowlesi*; these are mainly transmitted by the bite of a female mosquito from the genus *Anopheles* [3,4]. Transmission by becoming exposed to infected blood (blood transfusion) or congenital transmission have also been described, though occurring less frequently; most cases of malaria in industrialised nations involve travellers, immigrants or military personnel coming from endemic areas of the countries they have visited or lived in [4].

Most cases of malaria observed around the world are caused by *P*. *falciparum* and *P*. *vivax* [3,5]. A fourth (~26%) of the malaria-endemic areas worldwide are exposed to *P*. *falciparum* transmission, involving about one thousand million people [6]. *P*. *vivax* has a broader geographical reach than *P*. *falciparum*, covering at least 95 countries and involving tropical, sub-tropical and temperate regions, meaning that more people are exposed to infection by this parasite [5].

The distribution of *P*. *malariae* infection is rarely considered, however, its presence has been observed throughout all the world’s main endemic regions [7,8]. *P*. *malariae* is widespread in sub-Saharan Africa and the southeast of the Pacific region where its prevalence has surpassed 30% [8–10]; *P*. *malariae* cases are rare in South America [8,11,12], Central America [8,13], Asia [8,14–16] and the Middle East [8,17], having prevalence not above 2% [8].

There is little information about *P*. *malariae* in Colombia; infection prevalence (detected by PCR) has been reported to range from 10% to 20% in endemic areas of the Brazilian Amazon region and it has been observed that it circulates together with *P*. *brasilianum*, a frequent parasite in New World primates with which it shares 99% of its genetic information [8,18,19].

Zoonoses, particularly *P*. *vivax* and *P*. *malariae* sylvatic reservoirs in South America and Africa, can compromise malaria control and eradication efforts. This needs to be acknowledged by the public health authorities responsible for malaria control. In a broader context, the agencies responsible for health and environmental planning in the tropics need to be aware of present and likely future changes in levels of exposure to zoonotic malaria and develop appropriate mitigating and preventative strategies [20].

Malaria is currently considered one of the most serious public health problems in Colombia; more than 90% of the cases are limited to 70 municipalities (around 7% of all municipalities in Colombia), rural areas (85%) being the most affected by this disease [21]. *P*. *vivax* represents about 70% of the cases which are reported, whilst the remainder are attributed almost exclusively to *P*. *falciparum* [22]. There were no reports of cases of *P*. *malariae* malaria for 2015, according to the Colombian Public Health Surveillance System’s (SIVIGILA) epidemiological bulletins [23].

Under-registering infection prevalence for *P*. *malariae* and the low frequency reported for mixed infections could be partly due to thick smear limitations (i.e. of the gold standard for diagnosis). By contrast, molecular biology diagnostic techniques (conventional, nested and multiplex PCR) have been developed which even though they are not rapid and/or readily-accessible methods for diagnosing malaria in endemic areas, do have greater sensitivity, specificity and therefore can detect low parasitaemia (around five parasites /μL blood) [24].

Bearing in mind the restrictions of routine diagnosis tests for suitably identifying the different *Plasmodium* spp. species, the true prevalence of circulating parasites must be established. Correctly identifying the pathology’s causative agent is essential for treatment scheme administration and success, as well as strengthening and understanding infection dynamics aimed at adjusting public health programmes, considering the real panorama of the cases caused by this parasite [25].

This study was thus aimed at evaluating the infection and coinfection prevalence (simultaneous infection by multiple *Plasmodium* spp. species) of different *Plasmodium* species (*vivax*, *falciparum* and *malariae*) in a region of the Colombian Amazonian department. Under-registering of the prevalence reported for some species of *Plasmodium* spp. was found, as was a correlation between some factors associated with this disease. Such results portray a large-scale epidemiological problem concerning the correct diagnosis and subsequent treatment of the disease. Therefore, the present study represents a contribution towards knowledge regarding the dynamics of these infections in order to provide useful tools for strengthening existing measures for managing and preventing malaria.

## Methods

### Study population

**Fig 1:**
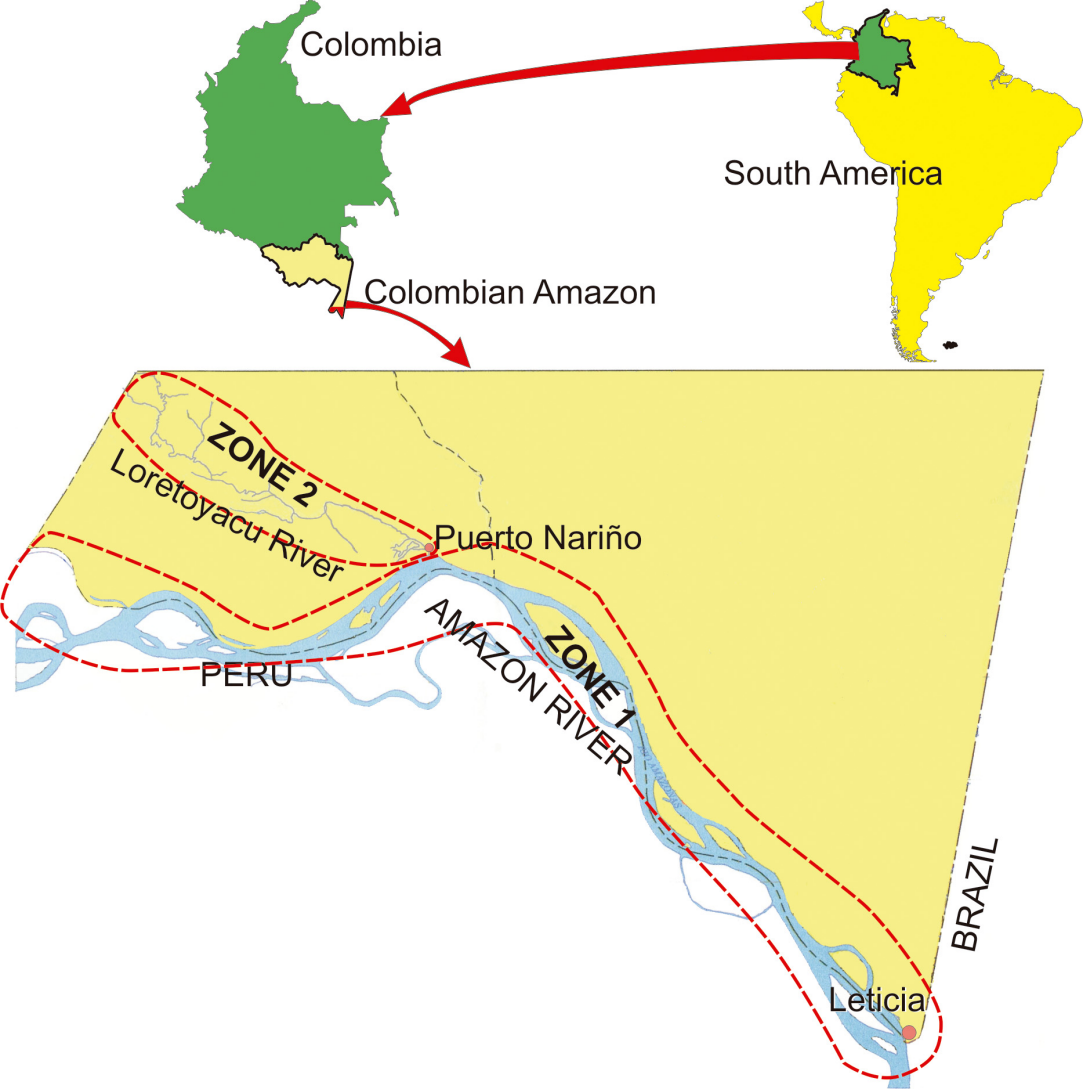
Geographical localisation of the population included in the study. The Colombian Amazon region and the Amazonas (zone 1) and Loretoyacu (zone 2) rivers, where this study took place, are portrayed.

The samples analysed in this study came from the municipalities of Leticia and Puerto Nariño in Colombia’s Amazonas department. Leticia has a projected population of 41,326 and Puerto Nariño 8,162 inhabitants, according to the Amazonas department’s development plan 2012–2015. The study took place in 53 settlements on the banks of the Amazon and Loretoyacu rivers which are located on the Amazonian frontier with Brazil and Peru (Fig 1, S1 Table).

### Sample size calculation and collection

This was a cross-sectional study; sample size was calculated using Epi Info 7 software, by assuming a 4.8% estimated prevalence in a similar population, having 5% significance level and a 95% confidence interval [26]. The minimum required sample size was thus 640 samples.

The inclusion criteria for obtaining samples took the following into account: patients who were symptomatic for malaria (headache, fever during the last 8 days, sweating, vomiting and/or diarrhoea), inhabitants living in the south of the Colombian Amazon region (previously described communities). The blood samples involved in this research were collected by Fundacion Instituto de Inmunología de Colombia (FIDIC) personnel between July and September 2015. Samples were obtained by skin puncture, collected on Flinders Technology Associates’ (FTA) cards and stored for subsequent *Plasmodium* spp. species’ detection by PCR.

### Ethics statement

Every individual signed an informed consent form after having received detailed information regarding the study’s objectives and answering a survey aimed at collecting sociodemographic characteristics. The informed consent form and survey were signed/filled out by a parent or tutor for patients aged less than 18 years-old and supervised by witnesses. This study was approved and supervised by the Universidad del Rosario’s School of Medicine and Health Sciences’ (EMCS) Research Ethics Committee (CEI) (Colombia: resolution CEI-ABN026-000161).

### Sample processing and molecular diagnosis of *Plasmodium* species

Genomic DNA samples were extracted from each drop of blood collected on the FTA cards using a Pure Link Genomic DNA mini kit (Invitrogen) according to the manufacturer’s specifications. The samples were eluted in a 50μL final volume of buffer containing 10 mM Tris-HCl, pH 9.0 and 0.1 mM EDTA. PCR was used for all samples, using primers directed at a segment of the human *β-globin* gene to guarantee the presence of DNA [27].

Samples proving positive by the PCR targeting the *β-globin* gene, were then submitted to nested PCR for identifying *Plasmodium* species; identification was done with specific primers for the parasite’s 18S ribosomal small subunit RNA (ssRNA) (S2 Table) [28]. The first PCR mixture contained 1X buffer, 3.8 mM MgCl_2_, 1.4 mM dNTPs, 0.2μM primers, 1U/μL Taq polymerase (Biolase DNA Polymerase, Bioline), 2μL genomic DNA and molecular grade water up to 21 μL final volume. Amplification conditions were: 95°C x 5 min, followed by 25 cycles at 94°C x 1 min, 58°C x 2 min and 72°C for 2 min and a final extension step at 72°C for 5 min.

The amplification product from the first PCR was used as template for a second PCR for type specific identification of *Plasmodium* spp. (*P*. *falciparum*, *P*. *vivax* and *P*. *malariae*), using specific internal primers for each species [28]. The conditions for the mixture used in this second PCR were: 1X buffer, 4 mM MgCl_2_, 2.5 mM dNTPs, 0.25 μM primers, 0.5 U/μL Taq polymerase, 2μL of the first PCR amplification product, and molecular grade water (for 20μL final volume). Amplification conditions were: 94°C x 5 min, followed by 35 cycles of 94°C x 30 sec, 58°C x 1 min and 72°C x 4 min with a final extension step at 72°C for 4 min.

DNA samples from the different *Plasmodium* spp. species (*P*. *falciparum*, *P*. *vivax* and *P*. *malariae*) were used as positive controls and ultrapure distilled water (Gibco) was used as negative control. All the products obtained were analysed on 2% agarose gels, stained with SYBER safe (Invitrogen) and visualised on a MiniBIS Pro (DNR Bio-Imaging Systems) image analyser. Considering the unexpectedly high prevalence of coinfection between *Plasmodium* spp. species found in the study, 10% of the second PCR products were randomly selected to be sent for sequencing in an ABI-3730 XL sequencer (Macrogen, Seoul, South Korea).

### Statistical analysis

Quantitative *Plasmodium* spp. species’ detection by PCR was reported as the mean and standard deviation (SD), whilst categorical variables were reported as percentages. Infection prevalence was presented in percentages with their respective 95% confidence intervals (CI). *Plasmodium* spp. species distribution was analysed according to geographical origin; Fisher’s exact test and χ^2^ were used for evaluating all the differences regarding percentages (according to the case). Variables such as age, gender, insecticide use, bednet use, geographical origin, having stagnant water nearby, the type of housing and clinical symptoms (fever, headache, vomiting, chills, diarrhoea or changes in urine) were treated as categorical variables.

The strong association between the variables was established using odds ratios (OR) with 95%CI. A theoretical direction was established for analysing them, where the dependent variables were the infecting *Plasmodium* spp. species (*P*. *falciparum*, *P*. *vivax* or *P*. *malariae*) and the state of infection (single, double- or triple-infection) whilst the independent variables became the categorical variables. The multivariate model was adjusted for age, gender, protection barriers (insecticide and bednet use), environmental factors (geographical origin, having stagnant water nearby, type of housing) and symptoms (fever, headache, vomiting, chills, diarrhoea or changes in urine). All hypothesis tests were fixed at 5% significance. Stata11 software was used for statistical procedures.

## Results

675 patients met the inclusion criteria and were thus invited to participate in the study; 4 of them were excluded as their samples could not be amplified for human β-globin. This gave 671 patients, aged 1 to 94 years-old (mean age 27.4; SD = 19.4), who were included in the statistical analysis; 51.1% (n = 343: 47.2–54.9 95%CI) of the study population were male and 48.9% female (n = 328: 45.0–52.7 95%CI).

The sociodemographic characteristics were categorised into two groups according to geographical location: zone 1 covered banks of the Amazon River and zone 2 the banks of the Loretoyacu River (Fig 1). The estimator revealed differences in some sociodemographic variables according to geographical area, such as access to public services (water, sewerage system, electricity supply and gas). Greater exposure to environmental factors (type of housing, having stagnant water nearby) associated with the risk of *Plasmodium* spp. infection was found for area 2 (Table 1).

**Table 1:** Demographic profile of the 671 patients who were symptomatic for malaria.

	On the banks of the Amazon River *n* = 344	On the banks of the Loretoyacu River *n* = 327	*p*
Age*	29.5 SD = 18.1	25.1 SD = 20.4	0.0017
	n (%)	n (%)	
**Gender**			0.858
Female	167 (50.9)	161 (49.1)	
Male	177 (51.6)	166 (48.4)	
**Access to public services**			
Water			<0.001
Yes	153 (74.3)	53 (25.7)	
No	191 (41.1)	274 (58.9)	
Sewerage system			<0.001
Yes	102 (81.6)	23 (18.4)	
No	242 (44.3)	304 (55.7)	
Electricity supply			0.022
Yes	320 (50.2)	317 (49.8)	
No	24 (70.6)	10 (29.4)	
Gas			<0.001
Yes	62 (93.9)	4 (6.1)	
No	282 (46.6)	323 (53.4)	
**Protection barriers**			
Insecticide use			<0.001
Yes	104 (65.0)	56 (35.0)	
No	240 (47.0)	271 (53.0)	
Bednet use			<0.001
Yes	296 (47.6)	326 (52.4)	
No	48 (98.0)	1 (2.0)	
**Environmental risk factors**			
Stagnant water near the housing			0.018
Yes	251 (48.7)	264 (51.3)	
No	93 (59.6)	63 (40.4)	
**Type of housing**			<0.001
Rural	280 (46.1)	327 (53.9)	
Urban	64 (100)	0 (0.0)	

* mean; SD = standard deviation

The percentages were calculated by rows

**Fig 2:**
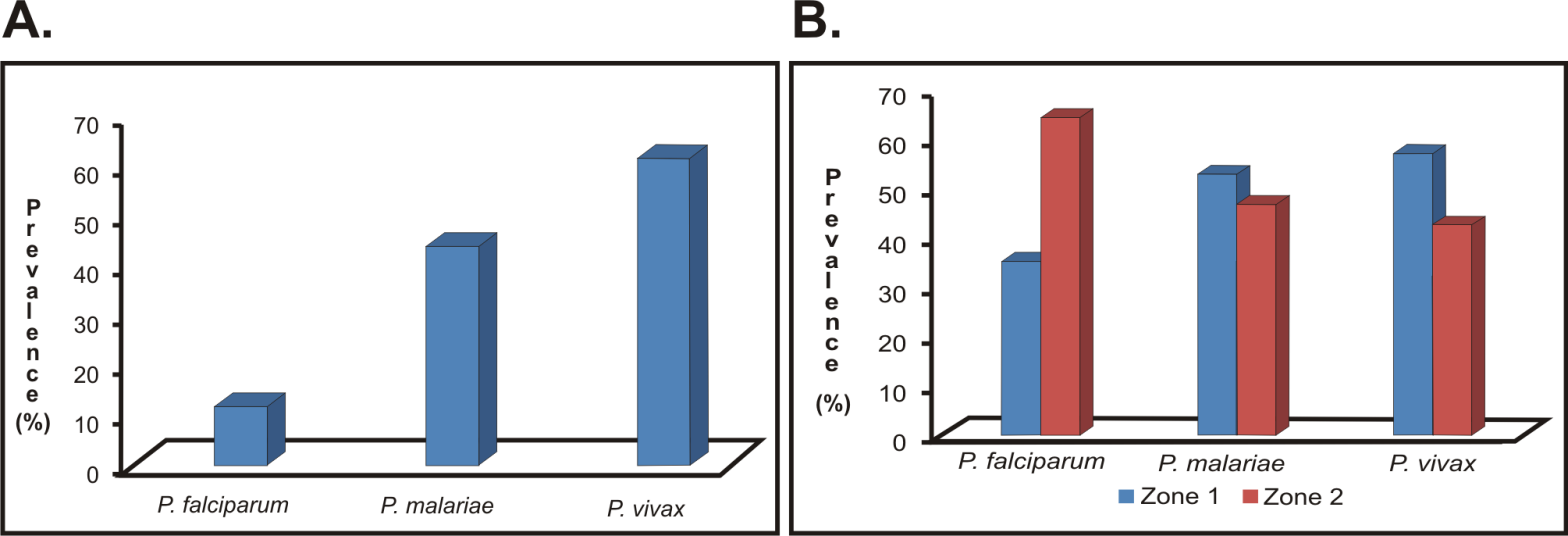
The prevalence of *Plasmodium* spp. infection. Overall prevalence in the population analysed. B. Overall prevalence by zone in which the analysed population were living.

Of all the samples analysed by PCR, 79.1% (n = 531: 75.8–82.1 95%CI) proved positive for *Plasmodium* spp.; regarding species distribution, *P*. *vivax* had the greater infection prevalence (61.4% of the infections; n = 412: 57.6–65.1 95%CI) followed by *P*. *malariae* (43.8%; n = 294: 40.0–47.6 95%CI) and then *P*. *falciparum* (11.8%; n = 79: 9.4–14.4 95%CI) (Fig 2A).

Regarding species distribution according to geographical origin, *P*. *vivax* was found to be the most prevalent species (57.3%; n = 236: 52.3–62.1 95%CI) whilst *P*. *falciparum* had the greatest species frequency for zone 2 (64.6%; n = 51: 52.9–74.9 95%CI). Such distributions were statistically significant (*p*<0.001). *P*. *malariae* distribution was similar in both areas, showing no statistically significant differences (*p =* 0.412) (Fig 2B).

**Fig 3:**
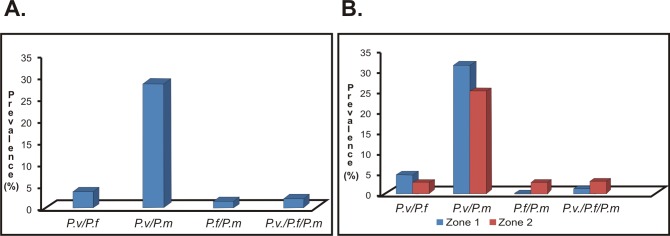
*Plasmodium spp*. coinfection prevalence. A. Prevalence of coinfection found in the population analysed. B. Prevalence of coinfection by area of the study population analysed. Abbreviations: *P*.*v/P*.*f*: *P*. *vivax/P*. *falciparum*, *P*.*v/P*.*m*: *P*. *vivax/P*. *malariae*, *P*.*f/P*.*m*: *P*. *falciparum/P*. *malariae*, *P*.*v/P*.*f/P*.*m*: *P*. *vivax/P*. *falciparum/P*. *malariae*.

Interestingly, high coinfection prevalence was found (defined as simultaneous infection by two or more different species of *Plasmodium* spp.) in the study population. Coinfection was found in 35.8% of the samples analysed (n = 240: 32.1–39.5 95%CI); the most frequently occurring combination was *P*. *vivax/P*. *malariae* (28.3%; n = 190: 24.9–31.8 95%CI), followed by *P*. *vivax/P*. *falciparum* (3.7%; n = 25: 2.4–5.4 95%CI) and *P*. *falciparum/P*. *malariae* (1.5%; n = 10: 0.7–2.7 95%CI). It was also found that 2.1% of the patients were infected by three *Plasmodium* spp. species (n = 14: 1.1–3.4 95%CI) (Fig 3A). *P*. *vivax/P*. *malariae* was the most frequently occurring combination in both areas analysed without statistically significant difference between them (*p* = 0.049). *P*. *falciparum/P*. *malariae* coinfection was greater in zone 2 than zone 1 (2.8%; n = 9: 1.2–5.1 95%CI). Triple infection was most prevalent in zone 2; however, the difference was not statistically significant (*p =* 0.107) (Fig 3B). Considering the unexpectedly high rate of coinfections, amplicons coming from 10% of the samples showing coinfection when analysed by PCR were sequenced and the diagnosis was confirmed in all of them.

After calculating the association between the different infections for each species with related factors (age, gender, insecticide use, bednet use, geographical origin, stagnant water nearby, type of housing and clinical symptoms), *P*. *vivax* was seen to have increased associations with slight (aOR: 1.60, 1.01–2.52 95%CI), moderate (aOR: 2.85, 1.62–5.01 95%CI) and severe headache (aOR: 5.38, 2.73–10.60 95%CI). By contrast, there were no changes in urine associated with infection by this parasite (amber aOR: 0.52, 0.34–0.79 95%CI) (intense brown aOR: 0.16, 0.07–0.36 95%CI) (Table 2).

**Table 2:** Factors associated with malarial infection for each *Plasmodium spp*. species.

Factors /Positive PCR	*P*. *vivax* n = (%)	aORs* (95%CI)	*P*. *falciparum* n = (%)	aORs* (95%CI)	*P*. *malariae* n = (%)	aORs* (95%CI)
Age*						
Less than 5 years old	42 (51.9)	Reference	8 (9.9)	Reference	27 (33.3)	Reference
5 to 18 years old	106 (63.1)	1.47 (0.82–2.62)	17 (10.1)	0.98 (0.39–2.44)	70 (41.7)	1.38 (0.77–2.45)
18 to 60 years old	237 (62.5)	1.09 (0.63–1.89)	48 (12.6)	1.73 (0.74–4.04)	176 (46.4)	1.56 (0.91–2.68)
Over 60 years old	27 (62.8)	1.49 (0.62–3.58)	6 (13.9)	1.53 (0.45–5.19)	21 (48.8)	**2.28 (1.01–5.15)**
**Gender**						
Male	212 (61.8)	Reference	46 (13.4)	Reference	147 (42.9)	Reference
Female	200 (61.0)	1.02 (0.72–1.44)	33 (10.1)	0.65 (0.39–1.07)	147 (44.8)	1.13 (0.81–1.56)
**Protection barriers**						
Insecticide use						
Yes	114 (71.3)	Reference	16 (10.0)	Reference	79 (49.4)	Reference
No	298 (58.3)	0.76 (0.48–1.18)	63 (12.3)	1.02 (0.54–1.93)	215 (42.1)	0.92 (0.62–1.37)
Bednet use						
Yes	377 (60.6)	Reference	76 (12.2)	Reference	268 (43.1)	Reference
No	35 (71.4)	0.88 (0.40–1.93)	3 (6.1)	0.98 (0.25–3.77)	26 (53.1)	1.38 (0.69–2.74)
**Environmental risk factors**						
Geographical origin						
Area 1	236 (68.6)	Reference	28 (8.1)	Reference	156 (45.4)	Reference
Area 2	176 (53.8)	0.93 (0.81–1.07)	51 (15.6)	**1.39 (1.12–1.72)**	138 (42.2)	1.02 (0.90–1.17)
Stagnant water nearby the housing						
No	304 (59.0)	Reference	67 (13.0)	Reference	216 (45.9)	Reference
Yes	108 (69.2)	1.23 (0.79–1.93)	12 (7.7)	0.66 (0.33–1.33)	78 (50.0)	1.21 (0.82–1.80)
Type of housing						
Urban	42 (65.6)	Reference	3 (4.7)	Reference	29 (45.3)	Reference
Rural	370 (60.9)	1.50 (0.75–3.00)	76 (12.5)	1.46 (0.38–5.59)	265 (43.7)	1.40 (0.75–2.64)
**Symptoms**						
Fever						
No	24 (42.9)	Reference	3 (5.4)	Reference	19 (33.9)	Reference
Yes	388 (63.1)	1.81 (0.94–3.48)	76 (12.4)	1.56 (0.44–5.46)	275 (44.7)	1.85 (0.97–3.53)
Headache						
No	64 (39.5)	Reference	23 (14.2)	Reference	53 (32.7)	Reference
Slight	124 (56.6)	**1.60 (1.01–2.52)**	28 (12.8)	0.86 (0.45–1.67)	89 (40.6)	1.10 (0.69–1.74)
Moderate	126 (73.3)	**2.85 (1.62–5.01)**	16 (9.3)	0.73 (0.31–1.70)	91 (52.9)	**1.77 (1.03–3.03)**
Severe	98 (83.1)	**5.38 (2.73–10.60)**	12 (10.2)	0.94 (0.37–2.36)	61 (51.7)	1.72 (0.95–3.10)
Vomiting						
No	330 (58.2)	Reference	66 (11.6)	Reference	235 (41.5)	Reference
Yes	82 (78.9)	1.57 (0.88–2.78)	13 (12.5)	1.52 (0.72–3.17)	59 (56.7)	**1.81 (1.12–2.92)**
Chills						
No	90 (55.9)	Reference	12 (7.5)	Reference	81 (50.3)	Reference
Yes	322 (63.1)	1.20 (0.78–1.85)	67 (13.1)	1.68 (0.84–3.34)	213 (41.8)	**0.58 (0.39–0.87)**
Diarrhoea						
No	378 (61.0)	Reference	75 (12.1)	Reference	274 (44.2)	Reference
Yes	34 (66.8)	0.63 (0.31–1.29)	4 (7.8)	0.63 (0.20–2.00)	20 (39.2)	0.53 (0.27–1.01)
Changes in urine						
Normal	196 (76.0)	Reference	26 (10.1)	Reference	131 (50.8)	Reference
Amber	203 (54.3)	**0.52 (0.34–0.79)**	45 (12.0)	0.94 (0.51–1.74)	151 (40.4)	0.93 (0.64–1.35)
Intense brown	13 (33.3)	**0.16 (0.07–0.36)**	8 (20.5)	2.45 (0.94–6.40)	12 (30.8)	**0.44 (0.20–0.94)**

*p* <0.05 values are indicated in bold.

*ORs adjusted for age, gender, protection barriers (insecticide and bednet use), environmental factors (geographical origin, stagnant water nearby, type of housing) and symptoms (fever, headache, vomiting, chills, diarrhoea, changes in urine).

Regarding *P*. *falciparum*, regression analysis revealed the association of risk of infection for the population located on the banks of the Loretoyacu River (area 2, aOR: 1.39, 1.12–1.72 95%CI); however, no other factor was observed to be associated with infection caused by this parasite (Table 2).

*P*. *malariae* infection had a strong association with patients over 60 years old (aOR: 2.28, 1.01–5.15 95%CI), as well as with symptoms such as moderate headache (aOR: 1.77, 1.03–3.03 95%CI) and vomiting (aOR: 1.81, 1.12–2.92 95%CI). By contrast, symptoms such as chills (aOR: 0.58, 0.39–0.87 95%CI) and changes in urine had little association with infection caused by this parasite (aOR: 0.44, 0.20–0.94 95%CI) (Table 2).

After calculating the association between the different infections (single-, double- and triple-infection) with the factors evaluated here, the results highlighted age as being an important associated variable. Regarding double infection, as age increased so did the association with this event (5 to 18 years old (aOR 1.98, 1.03–3.80 95%CI), 18 to 60 years old (aOR 2.09, 1.13–3.87 95%CI) and aged over 60 (aOR 2.65, 1.09–6.45 95%CI), unlike the same variable showing little association with cases of triple-infection (5 to 18 years old (aOR 0.02, 0.01–0.45 95%CI), 18 to 60 years old (aOR 0.07, 0.01–0.61 95%CI)) (Table 3).

**Table 3:** Factors associated with malarial coinfection according to the number of *Plasmodium spp*. species.

	Single infection n (%)	aORs* (95%CI)	Double infection n (%)	aORs* (95%CI)	Triple infection n (%)	aORs* (95%CI)
**Age**						
Aged less than 5 years old	33 (40.7)	Reference	16 (19.8)	Reference	4 (4.9)	Reference
5 to 18 years old	75 (44.6)	1.16 (0.67–2.01)	56 (33.3)	**1.98 (1.03–3.80)**	2 (1.2)	**0.02 (0.01–0.45)**
18 to 60 years old	165 (43.5)	1.12 (0.66–1.88)	139 (36.7)	**2.09 (1.13–3.87)**	6 (1.6)	**0.07 (0.01–0.61)**
Aged over 60	18 (41.9)	0.96 (0.43–2.13)	15 (34.9)	**2.65 (1.09–6.45)**	2 (4.7)	0.05 (0.03–6.70)
**Gender**						
Male	147 (42.9)	Reference	117 (34.1)	Reference	8 (2.3)	Reference
Female	144 (43.9)	1.03(0.75–1.41)	109 (33.2)	1.02 (0.72–1.43)	6 (1.8)	0.44 (0.09–1.99)
**Protection barriers**						
Insecticide use						
Yes	77 (48.1)	Reference	54 (33.7)	Reference	8 (5.0)	Reference
No	214 (41.9)	0.78 (0.53–1.16)	172 (33.7)	1.18 (0.78–1.79)	6 (1.2)	**0.15 (0.02–0.86)**
Bednet use						
Yes	270 (43.4)	Reference	206 (33.1)	Reference	13 (2.1)	Reference
No	21 (42.8)	0.69 (0.35–1.39)	20 (40.8)	1.25 (0.62–2.54)	1 (2.0)	3.53 (0.21–59.57)
**Environmental risk factors**						
Geographical origin						
Zone 1	154 (44.7)	Reference	127 (36.9)	Reference	4 (1.2)	Reference
Zone 2	137 (41.9)	0.98 (0.86–1.11)	99 (30.3)	0.95 (0.83–1.08)	10 (3.1)	**2.86 (1.40–5.84)**
Stagnant water nearby the housing						
No	77 (49.4)	Reference	173 (33.6)	Reference	9 (1.8)	Reference
Yes	214 (41.5)	1.32 (0.90–1.95)	53 (34.0)	0.94 (0.62–1.42)	5 (3.2)	3.43 (0.74–15.90)
Type of housing						
Urban	31 (48.4)	Reference	20 (31.2)	Reference	1 (1.6)	Reference
Rural	260 (42.8)	0.80 (0.43–1.49)	206 (33.9)	1.61 (0.82–3.13)	13 (2.1)	0.76 (0.04–14.23)
**Symptoms**						
Fever						
No	28 (50.0)	Reference	9 (16.1)	Reference	0 (0.0)	Reference
Yes	263 (42.8)	0.70 (0.38–1.30)	217 (35.3)	**2.64 (1.20–5.79)**	14 (2.3)	ND
Headache						
No	68 (41.9)	Reference	33 (20.4)	Reference	2 (1.2)	Reference
Slight	97 (44.3)	1.02 (0.65–1.60)	72 (32.9)	1.62 (0.97–2.70)	0 (0.0)	ND
Moderate	70 (40.7)	0.74 (0.43–1.25)	71 (41.3)	**2.45 (1.36–4.42)**	7 (4.1)	0.53 (0.05–5.56)
Severe	56 (47.5)	0.93 (0.52–1.66)	50 (42.4)	**2.45 (1.30–4.63)**	5 (4.2)	1.64 (0.11–24.24)
Vomiting						
No	241 (42.5)	Reference	186 (32.8)	Reference	6 (1.1)	Reference
Yes	50 (48.1)	1.00 (0.62–1.60)	40 (38.5)	1.15 (0.70–1.87)	8 (7.7)	**23.18 (3.35–160.46)**
Chills						
No	64 (39.7)	Reference	55 (34.2)	Reference	3 (1.9)	Reference
Yes	227 (44.5)	1.40 (0.94–2.09)	171 (33.5)	0.79 (0.52–1.21)	11 (2.2)	0.50 (0.07–3.42)
Diarrhoea						
No	262 (42.3)	Reference	213 (34.3)	Reference	13 (2.1)	Reference
Yes	29 (56.9)	1.80 (0.96–3.37)	13 (25.5)	**0.48 (0.23–0.98)**	1 (2.0)	0.05 (0.01–1.43)
Changes in urine						
Normal	123 (47.7)	Reference	103 (39.9)	Reference	8 (3.1)	Reference
Amber	156 (41.7)	0.75 (0.52–1.09)	117 (31.3)	0.95 (0.65–1.40)	3 (0.8)	0.35 (0.04–2.71)
Intense brown	12 (30.8)	**0.47 (0.22–0.99)**	6 (15.4)	**0.29 (0.11–0.74)**	3 (7.7)	**15.61 (1.52–159.63)**

*p*<0.05 values are indicated in bold

*aORs odds ratio adjusted, for age, gender, protection barriers (insecticide use and bednets), environmental factors (geographical origin, stagnant water nearby, type of housing), symptoms (fever, headache, vomiting, chills, diarrhoea, changes in urine). ND; not determined.

Clinical manifestations such as fever (aOR: 2.64, 1.20–5.79 95%CI), moderate (aOR: 2.45, 1.36–4.42 95% CI) and severe headache (aOR: 2.45, 1.30–4.63 95%CI) were associated with double-infection, whilst only the clinical variables vomiting (aOR: 23.18, 3.35–160.46 95%CI) and the intense brown colour of urine (aOR: 15.61, 1.52–159.63 95%CI) were seen to have an association with triple-infection (Table 3). Regarding the geographical region analysed, triple-infection was the only associated event (aOR: 2.86, 1.40–5.84 95%CI) (Table 3).

Adjusted OR were calculated for evaluating the association between pairs of *Plasmodium* spp. species; the results showed increased *P*. *malariae* infection frequency (aOR: 1.44, 1.01–2.06 95%CI) amongst *P*. *vivax-*infected patients; by contrast, infections involving *P*. *malariae* and *P*. *falciparum* were less associated (aOR 0.050, 0.29–0.86 95%CI) (Table 4).

**Table 4:** Number of infection pairs and odds ratios (OR) according to pairwise combinations of *Plasmodium* spp.

	*falciparum*		*malariae*	
Plasmodium species, OR	No n (%)	Yes n (%)	No n (%)	Yes n (%)
***vivax***				
No	220 (84.9)	39 (15.1)	169 (65.2)	90 (34.7)
Yes	372 (90.2)	40 (9.7)	208 (50.5)	204 (49.5)
Adjusted OR (95%CI)	0.64 (0.38–1.08)		**1.44 (1.01–2.06)**	
***malariae***				
No	322 (54.4)	270 (45.6)		
Yes	55 (69.6)	24 (30.4)		
Adjusted OR^b^ (95%CI)	**0.050 (0.29–0.86)**			

*p* <0.05 values are indicated in bold

b. OR, adjusted for age, gender, protection barriers (insecticide use and bednets), Environmental factors (geographical origin, stagnant water nearby, type of housing), symptoms (fever, headache, vomiting, chills, diarrhoea, changes in urine)

## Discussion

This has been the first study in Colombia which has sought to establish circulating *Plasmodium spp*. species’ prevalence in an endemic region of the Amazon by means of molecular diagnostic methods. The results showed that *P*. *vivax* was the causative agent for the greatest rate of infection in the population being analysed, thereby agreeing with previous reports for tropical, subtropical and temperate regions [5,6]. This parasite’s biological attributes, such as its ability to form hypnozoites as well as other geo-environmental conditions favouring its transmission and life-cycle, may be the cause for the endemicity of this type of malaria in many Latin-American countries [5,6,29].

Furthermore, variations in *Plasmodium spp*. species’ geographical distribution may be due to differences in genetic polymorphisms, underlying parasite drug resistance and host susceptibility, in addition to mosquito vector ecology and transmission seasonality [30]; hence, the presence and relevance of such factors must be addressed in future studies.

Interestingly, the results showed that *P*. *malariae* species represented the second causative agent for malaria in the target population, a distribution differing from reports regarding thick smear use in Colombia; in fact, no *P*. *malariae* cases were reported in the whole country in 2015 [23]. The *P*. *malariae* sporogonic cycle is the longest for *Plasmodium* spp. According to a study by the United Nations and IDEAM, Colombia will experience a 2.14°C temperature increase by 2100 due to the effects of climate change [31].

Increased *P*. *malariae* infection could be due to climate change affecting the region, this being mainly related to temperature rise and new weather conditions (rainfall and humidity). Such changes could reduce sporogonic cycle duration within competent vectors. It is known that a temperature increase from 20 to 28°C shortens the parasite’s sporogonic cycle within a vector from 30–35 days to 14 days. Reducing the sporogonic cycle increases vector viability and survival, thereby allowing it to increase the number of infective bites during its life-cycle [32]. Prevalence not exceeding 10% has been reported in Latin-America in endemic areas of the Amazon region [2,3]; however, greater than 40% infection frequency has been reported in countries like Indonesia (also having malaria-endemic areas) [9].

This discrepancy between *P*. *malariae* infection frequencies may be partly related to the fact that thick smear is used as the gold standard in most endemic areas and, given this test’s limitations (i.e. inter-observer sensitivity, mixed infection and poor detection regarding low parasitaemia), it is likely that this parasite is under-registered [33].

Many of the samples proving positive for *P*. *malariae* in this study came from double- and triple-mixed infections. Moreover, several reports where PCR-determined prevalence for mixed infection in which *P*. *malariae* has been involved, have included samples overlooked by local microscopists who examined standard thick smears on-site [11,16]. Consequently, the thick smear could be contributing to the under-registering of *P*. *malariae* by only the most prevalent species in a mixed infection-sample being recorded, as coinfection usually implies the dominance of one of the species in it, the other one having only a few parasitic forms [34]. Routine diagnosis for *P*. *malariae* could also be limited by species miss-indentification, since some ring forms become morphologically altered in red blood cell thick smear staining [35]. Molecular biology techniques thus represent an alternative which is aimed at increasing malarial diagnosis sensitivity and specificity [24,36].

Although the aforementioned *P*. *malariae* prevalence was unexpected, it is worth mentioning that its presence has been reported along with the occurrence of *Plasmodium brasilianum*; this parasite is commonly found in New World monkeys which, phylogenetically, is the same species as *P*. *malariae* which has naturally adapted to grow in these primates following human settlement of South America within the last 500 years [18].

The findings which have led to suggesting that *P*. *malariae* and *P*. *brasilianium* are in fact a single species concern the very high genetic identity between both parasites, differing just in a range expected to occur within a species. This would include the high similarity between their 18S sequences and the striking identities for *msp-1*, *dhfr*, cytochrome b and microsatellite DNA gene targets, whose single nucleotide polymorphisms (SNPs) are randomly distributed and as no distinctive marker has been identified so far [19,37,38,39]. Furthermore, there is the cross-reactivity and neutralization of *P*. *brasilianum* sporozoite infectivity of monkeys and vice versa by monoclonal antibodies against the *P*. *malariae* circumsporozoite protein (CSP) [19], as well as evidence of *in vitro* and naturally-acquired infection in humans with parasites termed as being *P*. *brasilianum* [19].

Consequently, primate and human populations co-habitation, as well as the plausible transmission of parasites due to both sharing a common vector, may play a causal role in the prevalence observed for this parasite species. Further studies aimed at assessing the prevalence of different *Plasmodium* species within the circulating vectors may provide some insight into the possible transmission from monkeys to humans and vice versa.

This study has revealed high mixed infection prevalence, mainly by *P*. *vivax/P*. *malariae*, which contrasts with other reports where coinfection did not exceed 30% [10,11]. Such cases could have been underestimated when diagnosed by routine techniques, due to the aforementioned limitations of the thick smear for accurately reporting double- and triple-infections.

So far, 40 of the 500 species of *Anopheles* spp. have been associated with the transmission of malaria around the world, Colombia having 9 malaria transmitters [37,38], proving the plasticity of malaria parasites when it comes to adapting to new vectors for colonising new host populations. Furthermore, multiple parasite species can be transmitted by a single vector and can also be associated with specific species of vectors; an example of this would be mixed infection by *P*. *falciparum/P*. *vivax* which has been found to be related to transmission by *Anopheles dirus* and triple-infection by *P*. *falciparum/P*. *vivax/P*. *malariae* by *Anopheles maculatus* [36].

The above highlights the importance of vector dynamics as a possible cause for the surprising prevalence of coinfection observed here, along with the odd ratios for *P*. *vivax* and *P*. *malariae* occurrence (Table 4). Nevertheless, positive associations between *P*. *malariae* and other *Plasmodium* parasites have been considered to represent more likely individual differences regarding exposure or susceptibility to infection, rather than true biological interactions between the parasite species [8]. Infection by multiple species is important as it modifies the intra-host dynamics of the plasmodia infecting humans and the corresponding clinical manifestations, thus influencing infection epidemiology [39].

Although *P*. *falciparum* has been classically associated with a more severe clinical spectrum, multiple studies worldwide have reported increasing occurrence of severe *P*. *vivax* infection, a relevant matter now on the malaria eradication agenda. This pattern has also been observed in Colombia in recent decades [40].

This study’s results represent an important change in the overall epidemiological landscape for the Colombian Amazonian region, therefore affecting the underlying knowledge from which the current strategies for malaria control are designed.

Regarding the Amazon region, where about 90% of positive diagnosis by thick smear is due to *P*. *vivax* infection [39], a therapeutic scheme for this region’s population includes an initial dose of 10 mg/Kg chloroquine phosphate, followed by 7.5 mg/Kg 24 and 48 hours later, as well as 0.25 mg/Kg/day primaquine for 14 days. There is a similar scheme for *P*. *malariae* infection, but without primaquine [41]. Infection due to *P*. *malariae* and mixed *P*. *vivax/P*. *malariae* infection could be covered by such treatment; however, current knowledge regarding the parasite’s susceptibility to the antimalarial drugs used in such schemes must be expanded due to the little that is known about the biology of *P*. *malariae* and its present under-registering. In accordance with the 3% *P*. *vivax*/*P*. *falciparum* coinfection rate found here, the use of an artemisin-based combination therapy (ACT) plus primaquine is recommended [42].

More *P*. *malariae* infection cases being found, mainly in patients over 60, could indicate that this species can remain in the body as long asymptomatic infection. *P*. *malariae* can cause prolonged asymptomatic infection which can become reactivated decades after initial infection and manifest as an indolent illness associated with insidious weight loss, splenomegaly, anaemia, and hypergammaglobulinaemia [43].

Considering the other combinations of mixed infections, it is paramount to define a reliable diagnostic strategy for the identification of the aetiological agent causing the disease to establish a control strategy which is species-targeted. Particularly concerning Colombia, mixed *P*. *falciparum*/*P*. *vivax* infection might lead to improper treatment for both species if either species is diagnosed alone, since falciparum circulating parasites could be resistant to first-line treatment and misdiagnosed vivax infections may not be receiving the full anti-hypnozoite treatment with primaquine. Issues such as *P*. *falciparum* resistance to chloroquine [44] highlight the importance of carrying out more studies concerning the disease’s transmission potential and dynamics as therapeutic schemes usually change according to this parasite’s unique attributes. The importance of suitable diagnosis for identifying *Plasmodium spp*. species has a direct influence on the clinical, epidemiological and pharmacological management of such infections [33]. A wrong diagnosis of malaria due to species misidentification or overlooking mixed-infections, could contribute towards the selective pressure of genotypes which are resistant to antimalarial drugs and thus lead to therapeutic failure [45].

The present study has evaluated different factors associated with infection by *Plasmodium spp*. species which could act as risk factors for developing malaria, as well as some related clinical manifestations. The results showed that age was a related factor; a peak in *P*. *malariae* infection was observed here in advanced age groups. This contrasts with previous reports in which *Plasmodium* infection is frequently associated with young populations [8]. Further studies assessing the relevance of age groups in *P*. *malariae* infection are recommended.

Regarding factors related to the clinical manifestations of *Plasmodium spp*. infection, the results correlated with symptoms such as headache, vomiting and changes in urine colour; in spite of studies highlighting some of these symptoms’ relationship with determined species [24,46–49], the infection’s clinical course is similar and no symptom by itself can predict a differential diagnosis for the infecting species [50].

An important remark considering the effectiveness of bednet and insecticide use is that no correlation was found between them and malaria infection incidence. This contrasts with the known effect of this practice in limiting transmission and thus contributing towards disease control [51,52]. Studies regarding vector response to such control measures should be performed, given previous reports suggesting vector resistance towards compounds present in insecticides [53].

The results from analysing the combination of infection by different *Plasmodium* spp. species showed positive associations for *P*. *vivax* and *P*. *malariae*. The additive effects of the presence of more than one species could increase the risk of developing aggressive clinical pictures of malaria [46]. This could also lead to relapses in the exposed population, as well as increased selective pressure from drug-resistant genotypes [47–49]. Nevertheless, the underlying biological and /or socio-demographic mechanisms for this relationship are yet to be determined.

This study’s cross-sectional design represents a limitation since it did not allow complete characterisation of the chronology of events related to coinfection and combinations involving infecting species, i.e. it did not lead to establishing whether these events occurred simultaneously or at different moments. Likewise, clinical features regarding the participants’ nutritional status were not taken into account.

In spite of the conventional treatment used for *P*. *vivax* also being able to be used for treating *P*. *malariae*, the latter still has high circulation in the Amazon trapezoid population; studies directed towards broadening knowledge of the natural history of the interactions between parasite-host aimed at establishing resistance patterns and infection dynamics are thereby needed. These should lead to effective prevention control measures and the drawing up of treatment measures for these species alone and in combination.

This study has revealed the high and important prevalence of infection and coinfection events which have possibly been under-registered to date. It has also contributed towards knowledge regarding the importance of the precise identification of the malarial parasite for correct clinical and epidemiological management. Future studies concerning vectors should be made, supporting the above conclusions drawn from the observations, as well as the ecological parasite–host relationship and environmental interactions to improve study design and control measures.

## Supporting Information

S1 Table A description of the communities included in the study for each geographical area. (DOCX)

S2 Table Primer sequences for *Plasmodium* species identification and amplicon size. (DOC)
