# Supplementary material for: High Plasmodium malariae Prevalence in an Endemic Area of the Colombian Amazon Region
Source: PLoS One. 2016 Jul 28;11(7):e0159968. doi: 10.1371/journal.pone.0159968 (PMC4965042; doi:10.1371/journal.pone.0159968)
Supplement: S1 Table — (DOCX) [file pone.0159968.s001.docx]

**S1 Table. A description of the communities included for each geographical area**

| Zone | Communities | | |
| --- | --- | --- | --- |
| 1 | La Libertad  Arara  Boyawuazú  Calderón  Km 11  Km 12  Km 18  Km 6  Km 6.5  Km 7  Km11  Km14  Km9  Yaguas  Isla de la Fantasía | La Milagrosa  La Playa  La Sarita  Loma Linda  Macedonia  Mocagua  Naranjales  San Martin de Amacayacu  San Sebastián de los Lagos  Santa Sofía  Tarapacá  Ticoya  Valencia  Canaan  Castañal los Lagos | Nazareth  Nuevo Jardín  Palmeras  Puerto Triunfo  Sambrano  San Antonio de los Lagos  San José de los Parentes  San José del Rio  San Juan de Atacuari  San Juan de los Parentes  El Progreso  El Vergel  El Calderón |
| 2 | San Francisco  Nuevo Paraíso  Santa Teresita  Villa Andrea  San Juan del Soco  12 de Octubre  Puerto Rico  Santarén  San Pedro de Tipisca |  |  |
