# Supplementary material for: High Plasmodium malariae Prevalence in an Endemic Area of the Colombian Amazon Region
Source: PLoS One. 2016 Jul 28;11(7):e0159968. doi: 10.1371/journal.pone.0159968 (PMC4965042; doi:10.1371/journal.pone.0159968)
Supplement: S2 Table — (DOC) [file pone.0159968.s002.doc]

**Table S2. Primer sequence and amplicon size as described by Snounou *et al*., 1993**

| **Primer name** | **Nucleotide sequence (5’- 3’)** | **Parasite targeted** | **Amplicon length (bp)** |
| --- | --- | --- | --- |
| rPLU 5 | CCTGTTGTTGCCTTAAACTTC | *Plasmodium sp*. (first amplification) | ~1200 |
| rPLU6 | TTAAAATTGTTGCAGTTAAAACG |
| rFAL 1 | TTAAACTGGTTTGGGAAAACCAAATATATT | *P. falciparum* | 205 |
| rFAL2 | ACACAATGAACTCAATCATGACTACCCGTC |
| rVIV 1 | CGCTTCTAGCTTAATCCACATAACTGATAC | *P. vivax* | 117 |
| rVIV2 | ACTTCCAAGCCGAAGCAAAGAAAGTCCTTA |
| rMAL1 | ATAACATAGTTGTACGTTAAGAATAACCGC | *P. malariae* | 144 |
| rMAL2 | AAAATTCCCATGCATAAAAAATTATACAAA |
